# Diaryl ethers with carboxymethoxyphenacyl motif as potent HIV-1 reverse transcriptase inhibitors with improved solubility

**DOI:** 10.1080/14756366.2017.1387542

**Published:** 2017-11-03

**Authors:** Tomasz Frączek, Rafał Kamiński, Agnieszka Krakowiak, Evelien Naessens, Bruno Verhasselt, Piotr Paneth

**Affiliations:** a Institute of Applied Radiation Chemistry, Lodz University of Technology, Lodz, Poland;; b Department of Bioorganic Chemistry, Centre of Molecular and Macromolecular Studies, Polish Academy of Sciences, Lodz, Poland;; c Department of Clinical Chemistry, Microbiology and Immunology, Ghent University, Ghent University Hospital, Ghent, Belgium

**Keywords:** NNRTI, reverse transcriptase, HIV, drug solubility

## Abstract

In search of new non-nucleoside reverse transcriptase inhibitors (NNRTIs) with improved solubility, two series of novel diaryl ethers with phenacyl moiety were designed and evaluated for their HIV-1 reverse transcriptase inhibition potentials. All compounds exhibited good to excellent results with IC_50_ at low micromolar to submicromolar concentrations. Two most active compounds (**7e** and **7 g**) exhibit inhibitory potency comparable or even better than that of nevirapine and rilpivirine. Furthermore, SupT1 and CD4^+^ cell infectivity assays for the most promising (**7e**) have confirmed its strong antiviral potential while docking studies indicate a novel binding interactions responsible for high activity.

## Graphical abstract

**Figure:**
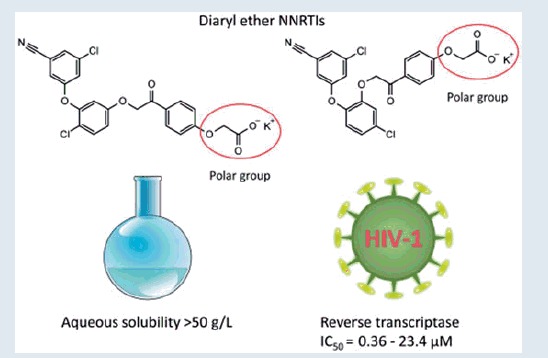

## Introduction

**Figure 1.**
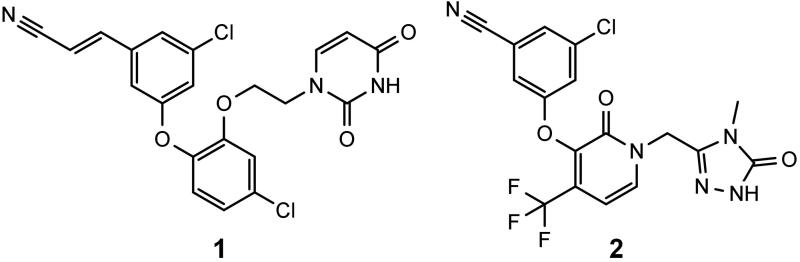
Structures of a catechol diether with the lowest EC_50_ reported to date (**1**) and doravirine (**2**).

Non-nucleoside reverse transcriptase inhibitors (NNRTIs) have proven their effectiveness as components of highly active antiretroviral therapy^1–3^. Their relatively low toxicity, as compared to other antiretroviral drugs, makes them a very attractive class of compounds used in treating HIV-1 infections^4–7^. Currently, there are five registered NNRTIs, first generation: nevirapine (NVP), efavirenz (EFV), delavirdine, and second generation: etravirine (ETV) and rilpivirine (RPV). Because HIV-1 reverse transcriptase (RT) has a low fidelity – its error rate was reported to be in the range of 10^−3^–10^−5^ per nucleotide addition^8–10^ – there is a very high mutation rate of the virus, and strains resistant to antiretroviral drugs emerge. Consequently, the pharmacotherapy may become ineffective, moreover, cross-resistance between NNRTIs is possible^11–14^. Another problem is that the NNRTIs binding site of RT favours non-polar compounds, which are usually poorly soluble in water. This is especially the case in second-generation NNRTIs, as both ETV and RPV are practically insoluble in water and require special formulations^15^ ^,^ ^16^. For these reasons there is a need to develop new NNRTIs with improved potency against resistant HIV mutants and better pharmacokinetics^17–19^. First generation NNRTIs like NVP and EFV are rigid molecules that bind well to the wild-type RT, but a single amino acid mutation in the binding site can significantly decrease their affinity to the enzyme. Second generation NNRTIs have flexible structures which allows them to adapt to a modified binding site of mutant RT^20^. Usually, second generation NNRTIs have 2–3 aromatic rings with an ether, thioether, short alkyl or amino group located between the rings that acts as a hinge that allows the inhibitors to bind in different conformations and overcome resistance mutations^20^ ^,^ ^21^. An excellent review on the chemical diversity of NNRTIs was written by Zhan et al.^18^. Diaryl ethers are one of the classes of second generation NNRTIs. There are several interesting inhibitors belonging to this class, including **1** – the most potent NNRTI reported to date (against wild type RT) and doravirine (**2**), which is in phase III clinical trials (Figure 1)^22–24^.

As mentioned above, poor solubility in water results in reduced bioavailability, and there is an increasing awareness of the need to design NNRTIs with improved pharmacokinetics. Several approaches were used by different authors to achieve better solubility of NNRTIs: salt formation^25^ ^,^ ^26^, prodrug formation^27^ ^,^ ^28^, addition of polar substituents^29–31^, modification of crystal structure^23^ or reduced halogenations^32^.

**Figure 2.**
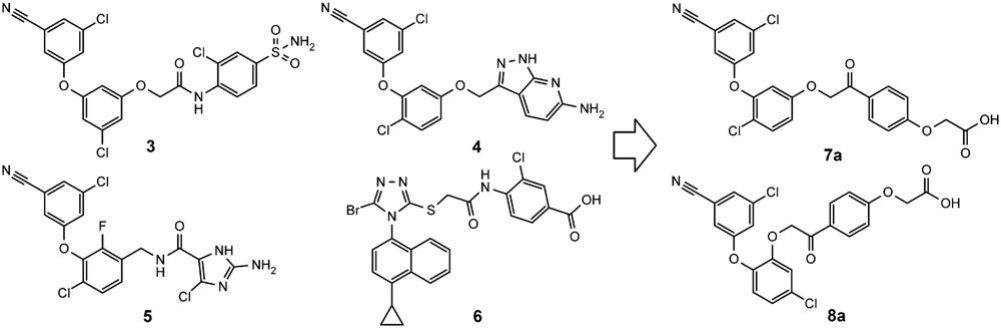
Structures of several diaryl ether NNRTIs (**3–5**), RDEA806 (**6**), and our newly designed compounds (**7a**, **8a**).

Our goal was to design second generation NNRTIs with improved solubility and chemical stability. Building on common substructures of several diaryl ether (**3**–**5**)^33–35^ and azole NNRTIs (**6**)^36^ we designed two new scaffolds: **7a** and **8a** (Figure 2). The new structures feature phenacyl moiety as an alternative to hydrolytically labile amide, found in some NNRTIs (Figure 2).

## Materials and methods

### Synthesis

**Figure 3.**
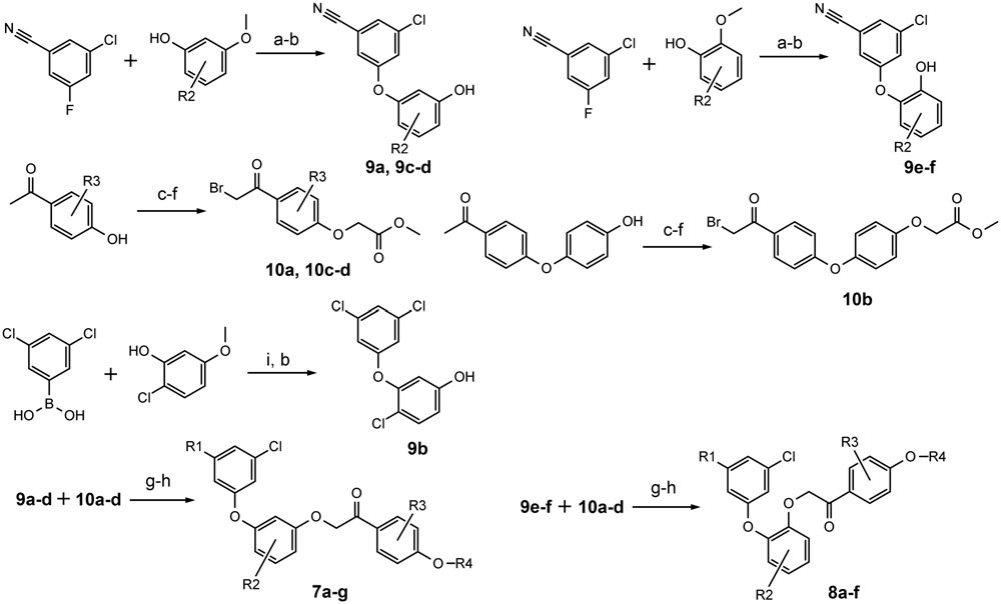
Synthesis scheme (a) K_2_CO_3_, N-methylpyrrolidone, 120 °C, 4 h (b) BBr_3_, CH_2_Cl_2_, 0–25 °C, 5 days (c) ethyl chloroacetate, K_2_CO_3_, KI, acetone, reflux, 4 h (d) NaOH, CH_2_Cl_2_ – CH_3_OH (9:1), 25 °C 1 h, then diluted HCl (e) CH_3_OH, *p*-toluenesulfonic acid, reflux, 4 h (f) N-bromosuccinimide, *p*-toluenesulfonic acid, CHCl_3_, 25 °C, 12 h (g) K_2_CO_3_, acetone, 25 °C, 4 h (h) K_2_CO_3_, CH_2_Cl_2_ – CH_3_OH – H_2_O, 25 °C, 1–2 days (i) Cu(CH_3_COO)_2_, pyridine, CH_2_Cl_2_, 25 °C, 2–3 days. R1-R4 groups are as in Table 1.

Compounds **7a**–**g** (resorcinol type) and **8a**–**f** (catechol type) were synthesised in several steps from commercially available starting materials. Diaryl ether parts (**9a**–**f**) of the new NNRTIs were synthesised from phenols and aryl fluorides in N-methylpyrrolidone (Figure 3) as described earlier^34^ ^,^ ^35^. In case of **9b** Chan-Lam coupling was used^37^. Hydroxyacetophenones were O-alkylated with ethyl chloroacetate. Subsequent exchange of ethyl to methyl afforded pure and solid methyl esters, which were selectively brominated with N-bromosuccinimide and *p*-toluenesulfonic acid in chloroform (**10a**–**d**) (Figure 3)^38^. Final deesterification was performed using potassium carbonate in a mixture of methylene chloride, methanol and water (room temperature, 1–2 days). Structures of obtained compounds are given in Table 1. Detailed synthetic procedures and characterisation data of reported compounds can be found in the supplemental material.

**Table 1.** Structures of synthesised compounds.

	
	R1	R2	R3	R4
**7a**	CN	2-Cl	H	CH_2_COOK
**7b**	Cl	2-Cl	H	CH_2_COOK
**7c**	CN	3-Cl	H	CH_2_COOK
**7d**	CN	H	H	CH_2_COOK
**7e**	CN	2-Cl	H	
**7f**	CN	2-Cl	2-CH_3_	CH_2_COOK
**7g**	CN	2-Cl	3-CH_3_	CH_2_COOK
**8a**	CN	4-Cl	H	CH_2_COOK
**8b**	CN	5-Cl	H	CH_2_COOK
**8c**	CN	4-Cl	H	
**8d**	CN	4-Cl	2-CH_3_	CH_2_COOK
**8e**	CN	5-Cl	3-CH_3_	CH_2_COOK
**8f**	CN	5-Cl	H	

### Molecular docking

The RCSB Protein Data Bank (PDB)^39^ contains over 150 crystallographic structures of HIV-1 reverse transcriptase-NNRTI complexes. Basing on structural similarity features, four PDB entries were selected for wild type RT receptor preparation: 3DRP, 4H4M, 3C6T and 2YNG (the structures of corresponding reference ligands can be found in the supplemental file, Fig. S1). RT mutant structures were taken from 3DRS, 3MEG (K103N), 3DRR, 1JLC (Y181C) and 4RW4, 3BGR (K103N/Y181C). In addition, 4G1Q structure was used as a wild type RT complex with RPV. Protein structures were prepared using Protein Preparation Wizard in Maestro^40^. After a preprocessing step with default settings, all waters, ions and small molecules were removed, hydrogen bond network was optimised, followed by global optimisation of all atoms. Receptor grids were prepared with default box size, centred on a reference ligand from the crystallographic structure. To simulate a small-scale adaptation of the receptor upon binding of a ligand, van der Waals radii of atoms with partial charges less than 0.2 were scaled down by a factor of 0.9^41^. Several docking protocols were used:

- Standard and extra precision (XP) Glide docking with default settings^42–45^. Three best poses were stored. For XP docking the threshold to reject a minimised pose was increased to 0.9 kcal/mol.
- Custom protocol 1 (CP1): The following positional constraint was added during receptor grid preparation – a 1 Å sphere centred at the oxygen atom of diphenyl ether motif of a reference ligand (from the crystallographic structure) had to be occupied by a neutral H-bond acceptor atom of a docked ligand. All ligand poses which did not comply with the constraint were rejected.
- Custom protocol 2: ligand poses obtained with the standard or custom (CP1) protocol were examined, and one best pose was manually selected (basing on the similarity to the reference ligand pose from crystallographic structure). All ligands were then aligned to the selected pose using the Flexible Ligand Alignment tool from Maestro, using a maximal common substructure. Subsequently, ligands were docked using Glide XP with the sampling mode set to “None (refine only)”

Only the best scoring pose obtained with either protocol was kept for each docked ligand. The complete results can be found in the supplemental file.

### IC_50_ measurements

HIV RT inhibitory activity of the new compounds was measured using the colorimetric Reverse Transcriptase Assay kit (Roche, Basel, Switzerland). The assay was performed according to the manufacturer’s instruction with the only modification that 2 ng (instead of suggested 4–6 ng which caused the reaction to run too fast) of enzyme supplied was used for the each reaction. Stock solutions of examined and reference compounds (NVP (TCI, Tokyo, Japan) and RPV) containing 5% dimethyl sulfoxide (Merck, Darmstadt, Germany) were used to prepare dilutions ranging from 0.1 to 50 µM. Averaged results from at least two measurements were used to obtain an inhibition curve. IC_50_ values were obtained by interpolating the curve using non-linear regression.

### Solubility

Substances were dissolved in ultrapure deionised water at room temperature up to concentrations of 50 g/L. Solutions were centrifuged and their aliquots were transferred into weighed glass vials (*d* = 0.01 mg). The samples were dried under reduced pressure and the vials were weighed again. The solubility was calculated from mass difference divided by sample volume.

### Infection assays and cytotoxicity measurements

CD4^+^ T cells were harvested from normal donors and cultured in Roswell Park Memorial Institute medium (RPMI) supplemented with 2 mM l-glutamine (Life Technologies, Merelbeke, Belgium), 10% (v/v) heat-inactivated foetal calf serum (Hyclone, Thermo Fisher Scientific, Waltham, MA), 100 U/mL penicillin and 100 µg/mL streptomycin (Life Technologies), 20 ng/mL interleukin-2 (IL-2; specific activity 10 U/ng, Peprotech, London, UK), and with 1 µg/mL phytohemagglutinin (PHA) mitogen (Thermo Fisher Scientific), 72 h prior to infection, as described earlier^46^. SupT1 cells were maintained at maximum 500,000 cells/mL, in Iscove’s Modified Dulbecco’s Medium (IMDM) supplemented with 2 mM l-glutamine (Life Technologies) and 10% (v/v) heat-inactivated foetal calf serum (Hyclone, Thermo Fisher Scientific, Waltham, MA), as described earlier^47^.

Lyophilised compound was dissolved in dimethyl sulfoxide to a concentration of 5 mM, and further diluted in cell culture medium (SupT1: IMDM and CD4^+^ T cells: RPMI). Cells were incubated 2 h prior infection in appropriate medium as described above, now also supplemented with diluted compound. Subsequently, cells (SupT1: 50,000 per 96 well, CD4^+^ T cells: 250,000 per 96 well) were infected with HIV (HIV NL4–3-GFP-I, an infectious virus expressing green fluorescent protein (GFP) from gfp-IRES-nef mRNA expressed from the nef locus, as described earlier^48^. Medium was as described above, now also supplemented with diluted compound (For CD4^+^ T cells, PHA was left out). Medium was refreshed after 24 h, keeping compound and supplement concentrations constant. After 72 h, cells were harvested, measured by flow cytometry for GFP expression and counted, using a MACSQuant flow cytometer (Miltenyi Biotec, Bergisch Gladbach, Germany). Infection rate measured by GFP expression was between 6 and 20%. Cell numbers were used to measure cytotoxicity (cell numbers in non-infected cultures supplemented with compound were compared to cell numbers obtained in parallel cultures without compound added: a reduction with more than 10% was considered to indicate cytotoxicity).

## Results and discussion

### RT inhibitory activity

**7a** and **8a** were tested for the inhibitory activity against HIV-1 RT. Both compounds were found to be NNRTIs with IC_50_ of 1.23 ± 0.05 μM and 23.4 ± 1.6 μM, respectively. Using **7a** and **8a** as lead compounds several of their analogues were prepared and tested *in vitro*. Replacement of nitrile with chlorine in **7b** was detrimental to the inhibitory activity (Table 2). Changing the position or removing the chlorine atom in the central ring of **7a** also resulted in increased IC_50_ values (**7c** and **7d**, Table 2), but in case of catechol ethers moving the position of chlorine from 4 in **8a** to 5 in **8b** was beneficial for activity (about seven-fold decrease the IC_50_ value, Table 2). Modifications introduced to the phenacyl moiety of the new inhibitors resulted in several interesting findings. **7e** was found to be the most active NNRTI in this study with IC_50_ 0.36 ± 0.01 µM, more potent than the drug NVP (IC_50_ = 0.75 ± 0.02 µM) and nearly as potent as RPV (IC_50_ = 0.32 ± 0.04 µM). Analogous modification in catechol series resulted in **8c**, more potent that its parent compound **8a** (IC_50_ 1.9 ± 0.11 µM). Methylation of **7a** in the phenacyl ring yielded **7f** (less potent) and **7 g**, which was found to be another potent inhibitor of HIV-1 RT, slightly more active than NVP (IC_50_ 0.65 ± 0.03 µM). Catechol analogues **8d-f** were found to possess comparable, low micromolar activity (Table 2).

**Table 2.** IC_50_ values, solubility and docking scores of examined compounds.

Compound	IC_50_ [µM]	Solubility [g/L]	Mean docking score
**7a**	1.23 ± 0.05	13.1	–14.94
**7b**	6.75 ± 0.50	4.3	–14.56
**7c**	8.30 ± 1.3	>50.0	–14.76
**7d**	9.78 ± 0.67	>50.0	–14.39
**7e**	0.36 ± 0.01	3.2	–17.07
**7f**	4.71 ± 0.43	8.9	–14.97
**7g**	0.65 ± 0.03	3.8	–15.14
**8a**	23.4 ± 1.6	>50.0	–14.85
**8b**	3.40 ± 0.30	>70.0	–14.73
**8c**	1.90 ± 0.11	>50.0	–16.60
**8d**	4.70 ± 0.70	14.4	–14.89
**8e**	3.07 ± 0.22	39.9	–15.30
**8f**	1.27 ± 0.05	>50.0	–16.59
**NVP**	0.75 ± 0.02	0.17^a^	–
**RPV**	0.32 ± 0.04	0.00002^b^	–

^a^Morelock et al.^49^

^b^Janssen et al.^15^.

### Molecular docking analysis

Using one of the three docking protocols, a good pose for every inhibitor could be found for all four receptors. Averaged docking scores are given in Table 2 (individual scores are in Table S4 of supplemental material). Interestingly, the obtained mean docking scores show quite good qualitative correlation with IC_50_ values (Table 2). For example, for compounds **7a**–**g** the docking scores almost correctly rank the inhibitors by their activity: **7e, 7 g, 7f, 7a, 7c, 7 b, 7d,** with only **7f** being swapped with **7a**, as well as **7c** with **7b**. For **8a**–**f** the docking scores also were able to identify the three most active inhibitors. The results show that the molecular docking may be a valuable tool in further development of NNRTIs from this chemical class.

**Figure 4.**
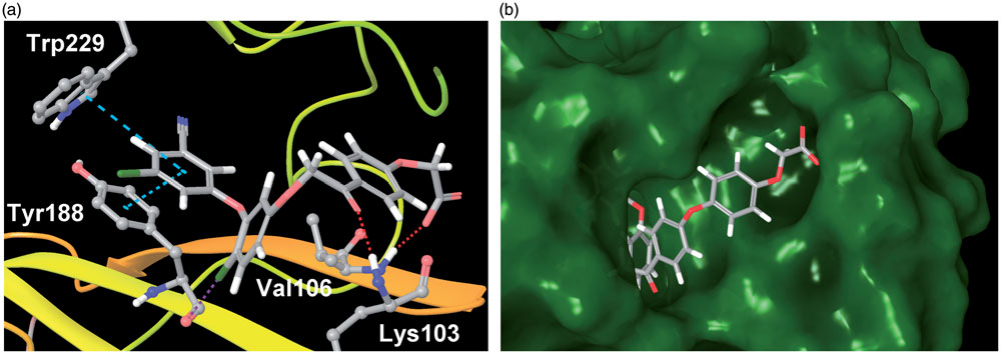
(a) Predicted binding mode of **7a** (PDB: 3C6T). Turquoise dashed lines – π–π stacking, red dashed lines – hydrogen bonds, purple dashed line – halogen bond. (b) Predicted binding mode of **7e** (PDB: 3C6T) viewed from the entrance to the NNRTIs binding site. Some residues removed for clarity.

The docking analysis was also useful in gaining some insight into the binding interactions of the new inhibitors with RT. The diaryl ether part of examined compounds binds in the hydrophobic cavity formed by non-polar aromatic amino acid residues of Tyr181, Tyr188, Phe227, Trp229 and Tyr318 (Figure 4(a)). The 3-chloro-5-cyanophenyl ring forms a π–π stacking interactions with Trp229 and Tyr188. The chlorine atom in the central aromatic ring points towards the carbonyl oxygen of Tyr188, forming a halogen bond. The carbonyl group of phenacyl moiety forms a hydrogen bond with the backbone oxygen of Lys103. Finally, the carboxyl group of three-ringed inhibitors locates itself at the solvent exposed entrance to the binding site, forming a hydrogen bond with Val106. In case of compounds **7e**, **8c** and **8f**, the additional phenyl ring between phenacyl and glycolic acid synthons is predicted to bind in an unexplored pocket adjacent to the entrance to the NNRTIs binding site (Figure 4(b)). This pocket may be an attractive target for the further optimisation of the new inhibitors. The predicted binding modes of **7e**, **8c** and **8f** suggest the existence of a strong ionic interaction between the inhibitors carboxyl and guanidine of Arg199, which explains the unusually high-docking scores for these three compounds (e.g. –19.62 for **7e** in 3C6T receptor, see Table S3), and may also be the cause of **7e** potency.

**Figure 5.**
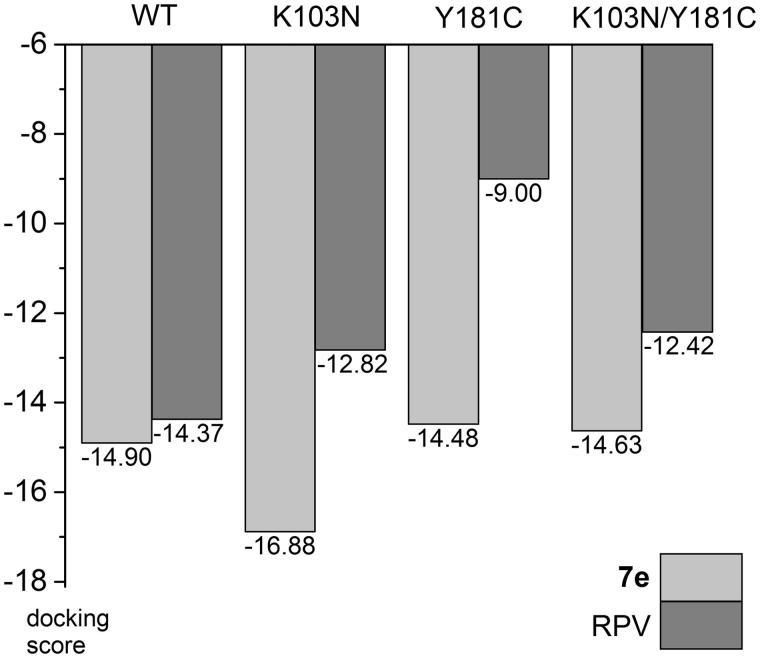
Comparison of docking scores of **7e** and RPV to selected RT mutants.

In order to assess expected activity of our best compound against mutated forms of the enzyme we have performed docking studies of **7e** and RPV – clinically used drug of the second generation that is active against these mutations. For wild-type, K103N, and Y181C forms 3DRP, 3DRS and 3DRR structures were chosen, respectively, in case of **7e**, because they share the same reference ligand. For K103N/Y181C double mutant 4RW4 structure was used. RPV was docked to 4G1Q (WT), 3MEG (K103N), 3JLC (Y181C) and 3BGR (K103N/Y181C), which all have RPV as the native ligand in crystal structures, with the exception of 3JLC, as there is no crystallographic data in PDB for RPV – Y181C complex. The results show a small decrease of docking scores for RPV in K103N and K103/Y181C, and a significant drop for Y181C mutant (in this case the decrease may be overestimated since this is not the native crystal structure, Figure 5). Interestingly, **7e** shows practically the same scores for WT, Y181C and K103N/Y181C forms, and an increased score for K103N (Figure 5). This suggests that **7e** is not sensitive to these mutations.

### Solubility

The aqueous solubility (at pH =7) of synthesised compounds is presented in Table 2. Many of the compounds show a good solubility, exceeding 50 g/L. Exact measurements of the maximum solubility proved to be infeasible due to the formation of micelles, aggregates and ultimately gels for increasing concentrations of the compounds. This behaviour results from the amphiphilic character of the examined compounds and elongated shapes of their molecules, which gives them surfactant-like properties. The general trend observed for all inhibitors is that catechol-based ethers (**8a**–**f**) are better soluble than their resorcinol counterparts (**7a**–**g**). Interestingly, removing or replacing the chlorine atom in two-position of resorcinol-based ethers like in case of **7c** and **7d** significantly increased their solubility. However, as discussed above, the two-position of the chlorine atom is optimal for the inhibitory activity. Methyl substitution in the phenacyl ring was also detrimental for the solubility. The most active compound **7e** shows relatively low solubility, but it is still ca. 100,000 times greater than that of RPV. Calculated octanol-water partition coefficients (logD) for examined compounds range from 0.97 to 3.05, which is close to that of NVP (2.49), and significantly lower than that of RPV (5.47, Table S5).

### Infection assays

First, toxicity of the compounds was determined in SupT1 cells. Since even minor toxicity affects the support of viral replication by the cell, compounds should be active well below threshold toxic concentration (i.e. concentration at which toxicity is observed in 10-fold titration series). The threshold toxic concentration, and whether antiviral activity was observed at 10-fold lower concentration than this threshold toxic concentration are given in Table 3.

**Table 3.** Threshold toxic concentrations and antiviral activity at 10-fold lower concentration of compounds examined.

Compound	Threshold toxicity [µM]	Antiviral activity
**7a**	5	–
**7b**	50	+
**7c**	50	–
**7d**	50	–
**7e**	50	+
**7f**	>50	+
**7g**	50	+
**8a**	50	–
**8b**	5	–
**8c**	50	+
**8d**	50	–
**8e**	50	+
**8f**	5	–
NVP	50	+
RPV	0.5	+

**Figure 6.**
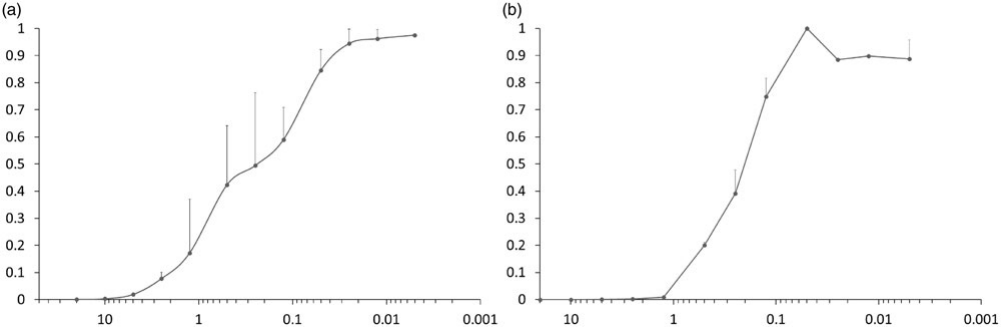
Cells were incubated with dilutions of **7e** and infected with HIV-1. Plots indicate concentration (in µM) vs. infection rate (normalised to level without inhibitor) for (a) SupT1 and (b) CD4^+^ T cells.

Several compounds tested in infectivity assay did not show antiviral activity at less than 10% of toxic concentration (Table 3). Nonetheless, of those who did, **7e** inhibited infection most clearly below concentrations which affected cellular viability. Therefore, this compound was tested more extensively in SupT1 cells, as well as in peripheral blood CD4^+^ cells. As shown in Figure 6, both in SupT1 cells as in primary T cells, IC_50_ was around 0.25 µM (toxicity was only apparent above 20 µM). The measurements were run in parallel with NVP as a reference, and it showed IC_50_ of 0.04 µM, in line with literature^50^.

## Conclusions

Two novel scaffolds of diaryl ether NNRTIs with phenacyl moiety were designed in this study. With the aid of molecular modelling several modifications of the core structures were prepared. Using molecular docking to several crystallographic structures and thorough conformational search of ligands, it was possible to obtain quite accurate predictions of structure–activity relationship. All synthesised compounds showed inhibitory activity against wild-type HIV-1 RT. In general, resorcinol-based compounds possessed better activities than catechol-based compounds, and are more promising candidates for further development. One of the compounds, **7e**, was found to be a very potent NNRTI in enzymatic assay, and is predicted to display novel interactions with the RT. The presented compounds were designed to possess a good aqueous solubility, which was achieved in all cases. Given those encouraging results, the inhibitors were subjected to biological evaluation of their efficacy against HIV infection *in vitro*. **7e** proved to be a potent anti-viral in SupT1 and CD4^+^ T cell infectivity assays. These results show our design could deliver highly water-soluble NNRTIs, at least one compound displays potent antiviral activity in infection assays *in vitro*.

## Supplementary Material

IENZ_1387542_Supplementary_Material.pdf
