# Supplementary material for: Diaryl ethers with carboxymethoxyphenacyl motif as potent HIV-1 reverse transcriptase inhibitors with improved solubility
Source: J Enzyme Inhib Med Chem. 2017 Nov 3;33(1):9–16. doi: 10.1080/14756366.2017.1387542 (PMC6009982; doi:10.1080/14756366.2017.1387542)
Supplement: IENZ_1387542_Supplementary_Material.pdf [file IENZ_A_1387542_SM7220.pdf]

Supporting material

Diaryl ethers with carboxymethoxyphenacyl motif as potent HIV-1 reverse transcriptase inhibitors with improved solubility

Tomasz Frączek[a], Rafał Kamiński[a], Agnieszka Krakowiak[a,b], Evelien Naessens[c], Bruno Verhasselt[c]#, Piotr Paneth[a]*

[a] Institute of Applied Radiation Chemistry, Lodz University of Technology, Zeromskiego 116, 90-924 Lodz, Poland

[b] Department of Bioorganic Chemistry, Centre of Molecular and Macromolecular Studies, Polish Academy of Sciences, Sienkiewicza 112, 90-363 Lodz, Poland

[c] Department of Clinical Chemistry, Microbiology and Immunology, Ghent University, Ghent University Hospital, De Pintelaan 182, B9000 Ghent, Belgium

#Correspondence to B. Verhasselt: Bruno.Verhasselt@UGent.be

*Correspondence to P. Paneth: piotr.paneth@p.lodz.pl

Synthesis. All reagents (Sigma Aldrich, TCI, Merck) and solvents (Chempur, Avantor Performance Materials) were of analytical grade purity and were used as received. NMR spectra were recorded using Bruker Avance spectrometers (250, 500 and 700 MHz). Mass spectra (ESI TOF) were recorded using Waters LCT Premier XE mass spectrometer (Milford, MA). Thin layer chromatography was performed with Merck 60F254 silica gel plates. Column chromatography was performed using Silica Gel 60 (230-400 mesh, Merck)

Compounds **9a** (3-chloro-5-(2-chloro-5-hydroxyphenoxy)benzonitrile), **9c** (3-chloro-5-(3-chloro-5-hydroxyphenoxy)benzonitrile), **9d** (3-chloro-5-(3-hydroxyphenoxy)benzonitrile), **9e** (3-chloro-5-(4-chloro-2-hydroxyphenoxy)benzonitrile) and **9f** (3-chloro-5-(5-chloro-2-hydroxyphenoxy)benzonitrile) were synthesised using a previously published method.[1,2] The physicochemical data of **9a**, **9c**, **9e**, and **9f** were reported elsewhere.[3,4]

4-chloro-3-(3,5-dichlorophenoxy)phenol (9f) 100 mg of 2-chloro-5-methoxyphenol, 394.1 mg of 3,5-dichlorophenylboronic acid (2.5 eq.) and 225.1 mg of copper(II) acetate (1.5 eq.) were suspended in methylene chloride, 166.4 µl of pyridine (2.5 eq.) were added and the mixture was stirred for two days. The mixture was filtered and washed several times with methylene chloride. The solid residue was discarded and combined liquids were concentrated. The residue was separated using column chromatography on silica gel (mobile phase: chloroform-hexane 1:7). The obtained product (1,3-dichloro-5-(2-chloro-5-methoxyphenoxy)benzene) was dissolved in methylene chloride and 4 molar equivalents of boron tribromide were added. The mixture was stirred at room temperature for 5 days. The reaction was quenched with water, washed several times with water and evaporated to dryness. The product was used without further purification. Yield 40%. ^{1}H NMR (250 MHz) (acetone-D_6) δ (ppm): 6.89-6.97 (m, 4H, ArH), 7.24 (dd, J=1.8, 1.8 Hz, 1H, ArH), 7.50 (dd, J=8.6, 0.5 Hz, 1H, ArH).

3-chloro-5-(3-hydroxyphenoxy)benzonitrile (**9d**) ^1H NMR (250 MHz) (C_6D_6) δ (ppm): 4.23 (s, 1H, OH), 6.20 (dd, J=2.3, 2.3 Hz, 1H, ArH), 6.30-6.38 (m, 2H, ArH), 6.70 (m, 2H, ArH), 6.87-6.96 (m, 2H, ArH); m.p. 105-107° C.

methyl 2-[4-(2-bromoacetyl)phenoxy]acetate (10a)

The following sequence of reactions was carried out without purification of intermediate products. 500 mg of 4-hydroxyacetophenone, 731 mg of KI (1.2 eq.) and 1015 mg of potassium carbonate (2 eq.) were suspended in acetone and refluxed for several minutes, then 590 µl of ethyl chloroacetate was added (1.5 eq.). The mixture was refluxed for 4 hours. The reaction mixture was extracted with methylene chloride and the solid residue was discarded. The liquid was concentrated to a colourless oil. The oil was dissolved in 15 ml of mixture of methanolic sodium hydroxide (2 eq. of NaOH) and methylene chloride (1:9) and stirred for 1 hour. The mixture was evaporated to dryness, dissolved in water and acidified with diluted HCl. The precipitate (2-(4-acetylphenoxy)acetic acid) was filtered off, washed and dried. The product was dissolved in an excess of methanol, and refluxed with a catalytic amount of anhydrous p-toluenesulfonic acid over molecular sieves (3Å) for several hours, until complete disappearance of starting material (monitored by TLC, chloroform-methanol 30:1). The reaction mixture was concentrated, diluted with methylene chloride, washed with sodium bicarbonate solution, then washed with water and evaporated to dryness. 400 mg of the product (methyl 2-(4-acetylphenoxy)acetate) was dissolved in chloroform and 410 mg (1.2 eq.) of N-bromosuccinimide and 73.1 mg (0.2 eq.) of p-toluenesulfonic acid monohydrate were added. The mixture was stirred in a dark place for several hours (>12) until all of the starting compound disappeared (controlled by TLC, chloroform-methanol 30:1). The mixture was washed with sodium bicarbonate solution, then water, dried, and concentrated under reduced pressure, layered

with n-heptane and placed in refrigerator. The precipitated product was washed with n-pentane and dried in vacuum. Yield over 4 steps: 50%. ^{1}H NMR (250 MHz) ($CDCl_3$) δ (ppm): 3.88 (s, 3H, CH_3), 4.45 (s, 2H, CH_2), 4.77 (s, 2H, CH_2), 7.02 (d, J=8.9 Hz, 2H, ArH), 8.03 (d, J=8.9 Hz, 2H, ArH); m.p. 65-67° C.

methyl 2-{4-[4-(2-bromoacetyl)phenoxy]phenoxy}acetate (**10b**) The same procedure as for **10a**, starting from 1-[4-(4-hydroxyphenoxy)phenyl]ethan-1-one. ^{1}H NMR (700 MHz) ($CDCl_3$) δ (ppm): 3.83 (s, 3H, CH_3), 4.39 (s, 2H, CH_2), 4.66 (s, 2H, CH_2), 6.95 (d, J=9.0 Hz, 2H, ArH), 6.97 (d, J=9.0 Hz, 2H, ArH), 7.03 (d, J=9.0 Hz, 2H, ArH), 7.96 (d, J=9.0 Hz, 2H, ArH); m.p. 79-82° C.

methyl 2-[4-(2-bromoacetyl)-3-methylphenoxy]acetate (**10c**) The same procedure as for **10a**, starting from 2-methyl-4-hydroxyacetophenone. ^{1}H NMR (700 MHz) ($CDCl_3$) δ (ppm): 2.58 (s, 3H, CH_3), 3.85 (s, 3H, CH_3), 4.41 (s, 2H, CH_2), 4.72 (s, 2H, CH_2), 6.80 (dd, J=8.7, 2.6 Hz, 1H, ArH), 6.83 (d, J=2.4 Hz, 1H, ArH), 8.03 (d, J=8.7 Hz, 1H, ArH); m.p. 66-68° C.

methyl 2-[4-(2-bromoacetyl)-2-methylphenoxy]acetate (**10d**) The same procedure as for **10a**, starting from 3-methyl-4-hydroxyacetophenone. ^{1}H NMR (250 MHz) (C_6D_6) δ (ppm): 2.23 (s, 3H, CH_3), 3.30 (s, 3H, CH_3), 3.86 (s, 2H, CH_2), 4.12 (s, 2H, CH_2), 6.23 (d, J=8.5, 1H, ArH), 7.26 (dd, J=8.5, 2.3 Hz, 1H, ArH), 7.69 (m, 1H, ArH); m.p. 108-110° C.

General synthetic procedure for 7a-g and 8a-f. 30 mg of phenol (**9a-f**) and 1.5 eq. of potassium carbonate were suspended in acetone and stirred for 30 minutes. 1.1 eq. of phenacyl bromide (**10a-d**) was added and the mixture was stirred for 4-5 hours at room temperature. The solvent was evaporated, the residue was extracted with methylene chloride, concentrated and layered with n-hexane. The product separated as colourless crystals or oil. For demethylation the

ester was dissolved in 3 ml of a mixture of methylene chloride – methanol (2:1) with a drop of water, and 2 eq. of potassium carbonate were added. The mixture was stirred for 1-2 days at room temperature. After the disappearance of the starting material (controlled by TLC, chloroform – methanol 20:1) the mixture was evaporated, dissolved in water and neutralized with a calculated amount of diluted hydrochloric acid. The precipitated product was washed several times with water and dried. The product was purified if necessary by column chromatography. Potassium salts were prepared by dissolving the compounds in methanol and adding 0.5 eq. of potassium carbonate.

Potassium 2-(4-{2-[4-chloro-3-(3-chloro-5-cyanophenoxy)phenoxy]acetyl}phenoxy)acetate (7a) ^{1}H NMR (700 MHz) (DMSO-d_6) δ (ppm): 4.18 (s, 2H, CH_2), 5.53 (s, 2H, CH_2), 6.89 (d, J=8.9 Hz, 2H, ArH), 6.97 (dd, J=8.9, 2.9 Hz, 1H, ArH), 7.00 (d, J=2.9 Hz, 1H, ArH), 7.36 (dd, J=2.0, 2.2 Hz, 1H, ArH), 7.44 (dd, J=2.3, 1.3 Hz, 1H, ArH), 7.53 (d, J=8.9 Hz, 1H, ArH), 7.79 (dd, J=1.8, 1.4 Hz, 1H ArH), 7.90 (d, J=8.9 Hz, 2H, ArH); ^{13}C NMR (125 MHz, CD_3OD) δ (ppm): 67.0, 70.2, 109.2, 113.5, 114.5, 114.5, 116.5, 117.8, 118.2, 121.3, 126.0, 127.0, 129.9, 131.1, 136.1, 150.3, 158.5, 158.6, 163.7, 174.1, 193.3; HRMS (ESI): *m/z* 472.0387 $[M+H]^+$ (Calculated for $C_{23}H_{16}Cl_2NO_6$: 472.0349); m.p. 204-205° C.

Potassium 2-(4-{2-[4-chloro-3-(3,5-dichlorophenoxy)phenoxy]acetyl}phenoxy)acetate (7b) ^{1}H NMR (700 MHz) (DMSO-d_6) δ (ppm): 4.12 (s, 2H, CH_2), 5.51 (s, 2H, CH_2), 6.85 (d, J=8.9 Hz, 2H, ArH), 6.93 (dd, J=8.9, 2.9 Hz, 1H, ArH), 6.94 (d, J=1.8 Hz, 2H, ArH), 6.98 (d, J=2.9 Hz, 1H, ArH), 7.33 (dd, J=1.8, 1.8 Hz, 1H, ArH), 7.50 (d, J=8.9 Hz, 1H, ArH), 7.86 (d, J=8.9 Hz, 2H, ArH); ^{13}C NMR (125 MHz, CD_3OD) δ (ppm): 67.0, 70.2, 109.1, 113.1, 114.5, 115.3, 117.9, 122.6, 127.0, 130.0, 130.9, 135.5, 150.8, 158.5, 158.6, 163.7, 174.2, 193.3; HRMS (ESI): *m/z* 480.969 $[M+H]^+$ (Calculated for $C_{22}H_{16}Cl_3O_6$: 481.0012); m.p. 217-218° C.

Potassium 2-(4-{2-[3-chloro-5-(3-chloro-5-cyanophenoxy)phenoxy]acetyl}phenoxy)acetate (**7c**) ^{1}H NMR (700 MHz) (DMSO-*d*$_6$) δ (ppm): 4.13 (s, 2H, CH_2), 5.54 (s, 2H, CH_2), 6.74 (dd, J=2.2, 2.2 Hz, 1H, ArH), 6.78 (dd, J=2.0, 2.0 Hz, 1H, ArH), 6.85 (d, J=9.0 Hz, 2H, ArH), 6.97 (dd, J=2.0, 2.0 Hz, 1H, ArH), 7.50 (dd, J=2.0, 2.0 Hz, 1H, ArH), 7.55 (dd, J=2.3, 1.3 Hz, 1H, ArH), 7.81 (dd, J=1.8, 1.4 Hz, 1H ArH), 7.88 (d, J=8.9 Hz, 2H, ArH); ^{13}C NMR (125 MHz, CD_3OD) δ (ppm): 67.0, 104.9, 111.8, 112.3, 114.5, 114.5, 114.7, 116.4, 120.1, 123.2, 126.7, 127.0, 130.0, 135.7, 136.1, 156.9, 158.1, 160.4, 163.7, 174.2, 193.1; HRMS (ESI): *m/z* 472.0388 $[M+H]^+$ (Calculated for $C_{23}H_{16}Cl_2NO_6$: 472.0349); m.p. 168-171° C.

Potassium 2-(4-{2-[3-(3-chloro-5-cyanophenoxy)phenoxy]acetyl}phenoxy)acetate (**7d**) ^{1}H NMR (700 MHz) (DMSO-*d*$_6$) δ (ppm): 4.13 (s, 2H, CH_2), 5.48 (s, 2H, CH_2), 6.66 (ddd, J=8.1, 2.3, 0.8 Hz, 1H, ArH), 6.76 (dd, J=2.3, 2.3 Hz, 1H, ArH), 6.83-6.86 (m, 3H, ArH), 7.32 (dd, J=8.2, 8.2 Hz, 1H, ArH), 7.38 (dd, J=2.2, 2.0 Hz, 1H, ArH), 7.45 (dd, J=2.3, 1.3 Hz, 1H, ArH), 7.76 (dd, J=1.8, 1.3 Hz, 1H ArH), 7.89 (d, J=8.9 Hz, 2H, ArH); ^{13}C NMR (125 MHz, CD_3OD) δ (ppm): 67.0, 70.0, 106.7, 111.4, 112.1, 114.5, 114.5, 116.5, 119.4, 122.5, 125.9, 127.1, 130.0, 130.6, 136.0, 156.1, 159.0, 160.0, 163.7, 174.1, 193.7; HRMS (ESI): *m/z* 438.0770 $[M+H]^+$ (Calculated for $C_{23}H_{17}ClNO_6$: 438.0744); m.p. 205-206° C.

Potassium 2-[4-(4-{2-[4-chloro-3-(3-chloro-5-cyanophenoxy)phenoxy]acetyl}phenoxy) phenoxy]acetate (**7e**) ^{1}H NMR (700 MHz) (DMSO-*d*$_6$) δ (ppm): 4.01 (s, 2H, CH_2), 5.55 (s, 2H, CH_2), 6.82 (d, J=9.0 Hz, 2H, ArH), 6.96 (dd, J=9.0, 2.9 Hz, 1H, ArH), 6.96-6.99 (m, 4H, ArH), 7.00 (d, J=2.9 Hz, 1H, ArH), 7.33 (dd, J=2.3, 1.9 Hz, 1H, ArH), 7.41 (dd, J=2.3, 1.3 Hz, 1H, ArH), 7.51 (d, J=9.0 Hz, 1H, ArH), 7.77 (dd, J=1.9, 1.3 Hz, 1H ArH), 7.97 (d, J=9.0 Hz, 2H, ArH); ^{13}C NMR (125 MHz, CD_3OD) δ (ppm): 67.4, 109.2, 113.5, 114.5, 115.7, 116.1, 116.5, 117.8, 118.2, 121.3, 121.3, 126.0, 128.2, 130.2, 131.1, 136.1, 148.2, 150.3, 156.2, 158.4, 158.5,

163.9, 175.0, 193.3; HRMS (ESI): *m/z* 564.0451 $[M+H]^+$ (Calculated for $C_{29}H_{20}Cl_2NO_7$: 564.0617); m.p. 204-205° C.

Potassium 2-(4-{2-[4-chloro-3-(3-chloro-5-cyanophenoxy)phenoxy]acetyl}-3-methyl-phenoxy)acetate (**7f**) ^{1}H NMR (700 MHz) (DMSO-d_6) δ (ppm): 2.37 (s, 3H, CH_3), 4.09 (s, 2H, CH_2), 5.40 (s, 2H, CH_2), 6.65-6.67 (m, 2H, ArH), 6.91-6.94 (m, 2H, ArH), 7.32 (dd, J=2.3, 1.9 Hz, 1H, ArH), 7.41 (dd, J=2.3, 1.3 Hz, 1H, ArH), 7.50 (dd, J=8.6, 0.5 Hz, 1H, ArH), 7.77 (dd, J=1.8, 1.3 Hz, 1H, ArH), 7.82 (d, J=9.5 Hz, 1H, ArH); ^{13}C NMR (125 MHz, CD_3OD) δ (ppm): 21.0, 66.9, 71.1, 109.2, 111.3, 113.5, 114.5, 116.5, 117.8, 118.2, 118.2, 121.2, 126.0, 126.2, 131.1, 131.4, 136.1, 142.5, 150.3, 158.5, 158.6, 162.1, 174.3, 195.7; HRMS (ESI): *m/z* 486.0522 $[M+H]^+$ (Calculated for $C_{24}H_{18}Cl_2NO_6$: 486.0511); m.p. 147-150° C.

Potassium 2-(4-{2-[4-chloro-3-(3-chloro-5-cyanophenoxy)phenoxy]acetyl}-2-methyl-phenoxy)acetate (**7g**) ^{1}H NMR (700 MHz) (DMSO-d_6) δ (ppm): 2.18 (s, 3H, CH_3), 4.16 (s, 2H, CH_2), 5.49 (s, 2H, CH_2), 6.73 (d, J=8.7 Hz, 1H, ArH), 6.94 (dd, J=8.9, 2.9 Hz, 1H, ArH), 6.96 (d, J=2.9 Hz, 1H, ArH), 7.33 (dd, J=2.3, 1.9 Hz, 1H, ArH), 7.41 (dd, J=2.3, 1.3 Hz, 1H, ArH), 7.50 (d, J=8.9 Hz, 1H, ArH), 7.72 (m, 1H, ArH), 7.75 (dd, J=8.6, 2.3 Hz, 1H, ArH), 7.77 (dd, J=1.8, 1.3 Hz, 1H, ArH); ^{13}C NMR (125 MHz, CD_3OD) δ (ppm): 15.2, 67.3, 70.1, 109.2, 110.5, 113.5, 114.5, 116.5, 117.8, 118.2, 121.3, 126.0, 126.5, 127.4, 127.9, 130.1, 131.1, 136.1, 150.3, 158.4, 158.6, 162.1, 174.4, 193.5; HRMS (ESI): *m/z* 486.0779 $[M+H]^+$ (Calculated for $C_{24}H_{18}Cl_2NO_6$: 486.0511); m.p. 201-203° C.

Potassium 2-(4-{2-[5-chloro-2-(3-chloro-5-cyanophenoxy)phenoxy]acetyl}phenoxy)acetate (**8a**) ^{1}H NMR (700 MHz) (DMSO-d_6) δ (ppm): 4.17 (s, 2H, CH_2), 5.63 (s, 2H, CH_2), 6.88 (d, J=8.9 Hz, 2H, ArH), 7.08 (dd, J=8.6, 2.3 Hz, 1H, ArH), 7.26 (d, J=8.6 Hz, 1H, ArH), 7.28 (d,

J=2.4 Hz, 1H, ArH), 7.39 (dd, J=2.3, 2.0 Hz, 1H, ArH), 7.44 (dd, J=2.4, 1.3 Hz, 1H, ArH), 7.70 (dd, J=1.8, 1.3 Hz, 1H ArH), 7.85 (d, J=8.9 Hz, 2H, ArH); ^{13}C NMR (125 MHz, CD_3OD) δ (ppm): 67.0, 70.3, 114.1, 114.4, 114.9, 116.7, 117.9, 120.9, 121.5, 123.5, 125.2, 127.0, 129.9, 131.5, 135.7, 141.3, 150.9, 159.2, 163.6, 174.1, 192.4; HRMS (ESI): *m/z* 472.0381 $[M+H]^+$ (Calculated for $C_{23}H_{16}Cl_2NO_6$: 472.0349); m.p. 155-156° C.

Potassium 2-(4-{2-[4-chloro-2-(3-chloro-5-cyanophenoxy)phenoxy]acetyl}phenoxy)acetate (**8b**) ^{1}H NMR (700 MHz) (DMSO-d_6) δ (ppm): 4.12 (s, 2H, CH_2), 5.56 (s, 2H, CH_2), 6.83 (d, J=9.0 Hz, 2H, ArH), 7.08 (d, J=9.0 Hz, 1H, ArH), 7.26 (dd, J=8.9, 2.6 Hz, 1H, ArH), 7.35 (d, J=2.6 Hz, 1H, ArH), 7.39 (dd, J=2.3, 1.9 Hz, 1H, ArH), 7.44 (dd, J=2.4, 1.3 Hz, 1H, ArH), 7.69 (dd, J=1.7, 1.4 Hz, 1H ArH), 7.82 (d, J=8.9 Hz, 2H, ArH); ^{13}C NMR (125 MHz, CD_3OD) δ (ppm): 67.0, 70.3, 114.1, 114.4, 115.4, 116.7, 118.0, 121.0, 122.6, 125.4, 125.9, 126.3, 127.0, 129.9, 135.7, 142.9, 149.3, 159.0, 163.6, 174.1, 192.6; HRMS (ESI): *m/z* 472.0411 $[M+H]^+$ (Calculated for $C_{23}H_{16}Cl_2NO_6$: 472.0349); m.p. 154-155° C.

Potassium 2-[4-(4-{2-[5-chloro-2-(3-chloro-5-cyanophenoxy)phenoxy]acetyl}phenoxy) phenoxy]acetate (**8c**) ^{1}H NMR (700 MHz) (DMSO-d_6) δ (ppm): 4.02 (s, 2H, CH_2), 5.62 (s, 2H, CH_2), 6.82 (d, J=9.0 Hz, 2H, ArH), 6.95 (d, J=9.0 Hz, 2H, ArH), 6.98 (d, J=9.0 Hz, 2H, ArH), 7.07 (dd, J=8.5, 2.4 Hz, 1H, ArH), 7.24 (d, J=8.5 Hz, 1H, ArH), 7.30 (d, J=2.4 Hz, 1H, ArH), 7.35 (dd, J=2.3, 1.9 Hz, 1H, ArH), 7.40 (dd, J=2.4, 1.3 Hz, 1H, ArH), 7.67 (dd, J=1.8, 1.3 Hz, 1H ArH), 7.91 (d, J=8.9 Hz, 2H, ArH); ^{13}C NMR (125 MHz, CD_3OD) δ (ppm): 67.4, 70.5, 114.1, 114.9, 115.7, 116.0, 116.7, 117.8, 120.9, 121.3, 121.6, 123.5, 125.2, 128.2, 130.2, 131.5, 135.7, 141.2, 148.2, 150.9, 156.2, 159.2, 163.8, 175.0, 192.4; HRMS (ESI): *m/z* 564.0582 $[M+H]^+$ (Calculated for $C_{29}H_{20}Cl_2NO_7$: 564.0617); m.p. 165-168° C.

Potassium 2-(4-{2-[5-chloro-2-(3-chloro-5-cyanophenoxy)phenoxy]acetyl}-3-methyl-phenoxy)acetate (**8d**) ^{1}H NMR (700 MHz) (DMSO-d_6) δ (ppm): 2.38 (s, 3H, CH_3), 4.21 (s, 2H, CH_2), 5.50 (s, 2H, CH_2), 6.66-6.71 (m, 2H, ArH), 7.05 (dd, J=8.6, 2.4 Hz, 1H, ArH), 7.22-7.25 (m, 2H, ArH), 7.38 (dd, J=2.3, 1.9 Hz, 1H, ArH), 7.42 (dd, J=2.3, 1.3 Hz, 1H, ArH), 7.66 (dd, J=1.8, 1.3 Hz, 1H, ArH), 7.79 (d, J=8.7 Hz, 1H, ArH); ^{13}C NMR (125 MHz, CD_3OD) δ (ppm): 20.8, 66.4, 71.2, 111.2, 114.0, 114.7, 116.7, 117.8, 118.0, 120.9, 121.4, 123.5, 125.2, 126.4, 131.4, 131.5, 135.6, 141.2, 142.4, 150.9, 159.2, 161.8, 173.7, 195.0; HRMS (ESI): *m/z* 486.0534 $[M+H]^+$ (Calculated for $C_{24}H_{18}Cl_2NO_6$: 486.0511); m.p. 100-101° C.

Potassium 2-(4-{2-[5-chloro-2-(3-chloro-5-cyanophenoxy)phenoxy]acetyl}-2-methyl-phenoxy)acetate (**8e**) ^{1}H NMR (700 MHz) (DMSO-d_6) δ (ppm): 2.16 (s, 3H, CH_3), 4.16 (s, 2H, CH_2), 5.54 (s, 2H, CH_2), 6.72 (d, J=8.6 Hz, 1H, ArH), 7.06 (d, J=9.0 Hz, 1H, ArH), 7.26 (dd, J=8.9, 2.6 Hz, 1H, ArH), 7.35 (d, J=2.6 Hz, 1H, ArH), 7.39 (dd, J=2.2, 2.0 Hz, 1H, ArH), 7.43 (dd, J=2.3, 1.3 Hz, 1H, ArH), 7.67-7.71 (m, 3H, ArH); ^{13}C NMR (125 MHz, CD_3OD) δ (ppm): 15.2, 67.3, 110.4, 114.1, 115.5, 116.7, 118.0, 121.0, 122.6, 125.4, 125.9, 126.3, 126.5, 127.3, 127.9, 130.1, 135.7, 142.9, 149.3, 159.0, 162.0, 174.4, 192.8; HRMS (ESI): *m/z* 486.0762 $[M+H]^+$ (Calculated for $C_{24}H_{18}Cl_2NO_6$: 486.0511); m.p. 117-118° C.

Potassium 2-[4-(4-{2-[4-chloro-2-(3-chloro-5-cyanophenoxy)phenoxy]acetyl}phenoxy) phenoxy]acetate (**8f**) ^{1}H NMR (700 MHz) (DMSO-d_6) δ (ppm): 4.02 (s, 2H, CH_2), 5.57 (s, 2H, CH_2), 6.82 (d, J=9.0 Hz, 2H, ArH), 6.95 (d, J=8.9 Hz, 2H, ArH), 6.98 (d, J=9.0 Hz, 2H, ArH), 7.12 (d, J=9.0 Hz, 1H, ArH), 7.27 (dd, J=8.9, 2.6 Hz, 1H, ArH), 7.36 (d, J=2.6 Hz, 1H, ArH), 7.37 (dd, J=2.3, 2.0 Hz, 1H, ArH), 7.42 (dd, J=2.3, 1.3 Hz, 1H, ArH), 7.69 (dd, J=1.7, 1.4 Hz, 1H ArH), 7.90 (d, J=8.9 Hz, 2H, ArH); ^{13}C NMR (125 MHz, CD_3OD) δ (ppm): 67.4, 114.1, 115.4, 115.7, 116.0, 116.6, 117.9, 120.9, 121.3, 122.7, 125.4, 126.0, 126.3, 128.2, 130.2, 135.7, 142.8,

148.2, 149.2, 156.2, 159.0, 163.8, 175.0, 192.6; HRMS (ESI): *m/z* 564.0907 $[M+H]^+$ (Calculated for $C_{29}H_{20}Cl_2NO_7$: 564.0617); m.p. 151-153° C.

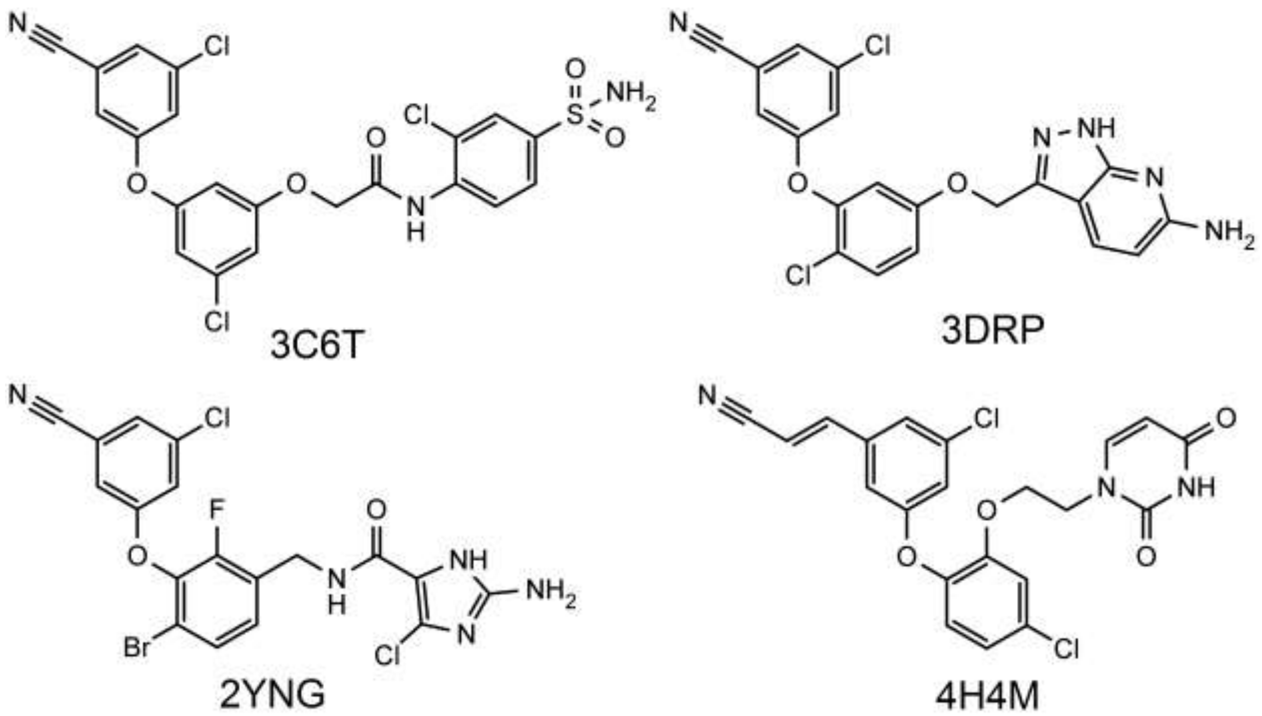

Figure S1. Structures of reference ligands from crystallographic structures used for receptor preparation for molecular docking**.**

Table S1. Mean docking scores for phenacyl substituted analogues of **7a** (core 1) and **8a** (core 2).

Core 1

R1= 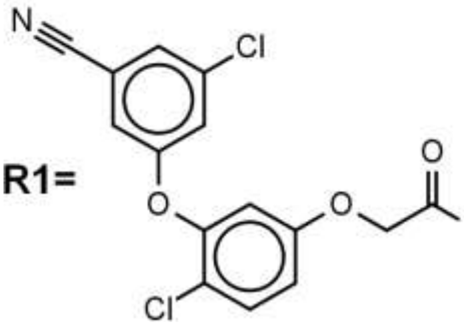

Core 2

R1= 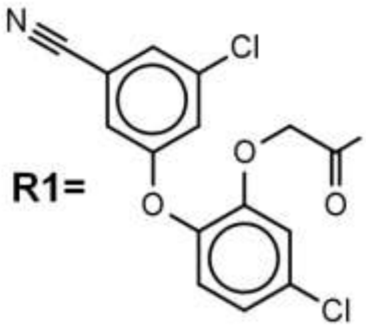

R2= OCH_2COOH

	Structure	Core 1	Core2
1	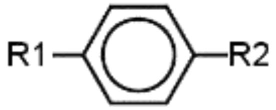	-14.94	-14.85
2	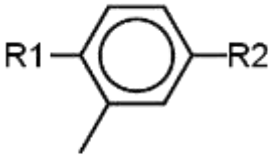	-14.97	-14.89
3	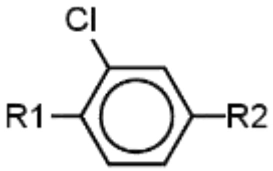	-14.61	-14.15
4	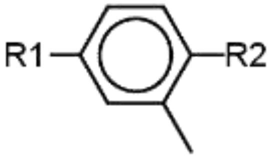	-15.14	-15.30

5	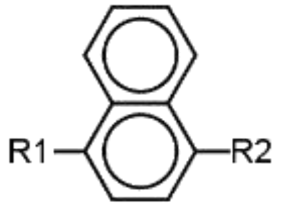	-14.79	-14.41
6	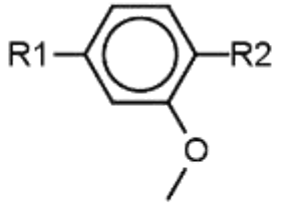	-14.83	-14.24
7	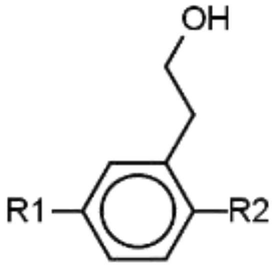	-15.70	-15.14
8	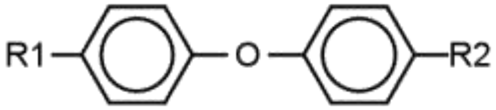	-17.07	-16.60
9	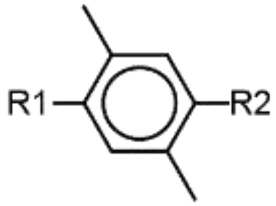	-14.55	-14.71
10	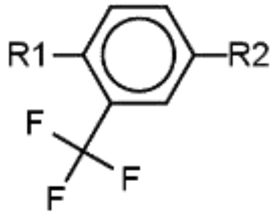	-14.74	-14.60

11	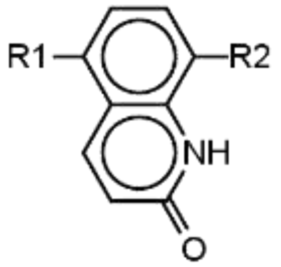	-13.68	-13.84
12	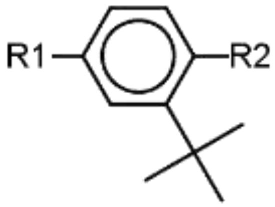	-13.38	-13.39
13	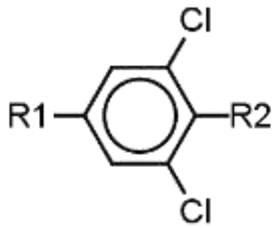	-14.61	-14.89
14	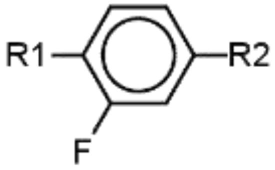	-14.47	-14.94
15	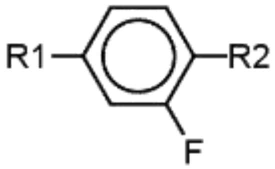	-15.09	-14.94

16	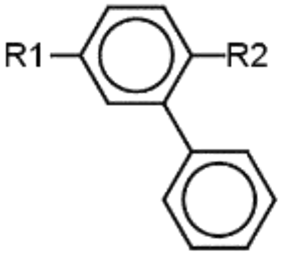	-14.25	-13.67
17	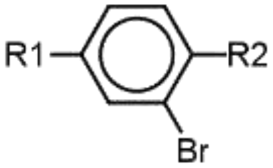	-15.03	-14.79
18	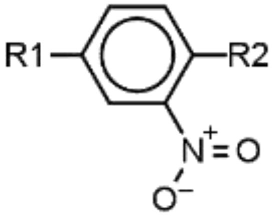	-14.53	-14.83
19	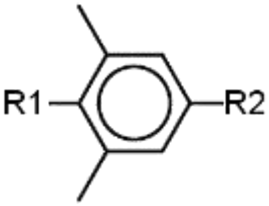	-13.25	-13.30
20	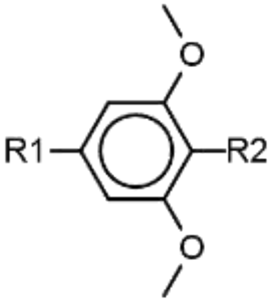	-14.67	-14.79

21	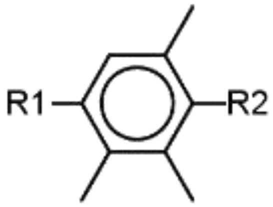	-15.11	-15.11
22	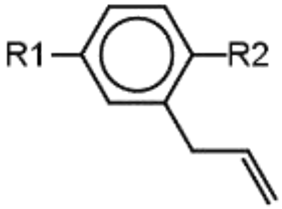	-14.84	-14.64
23	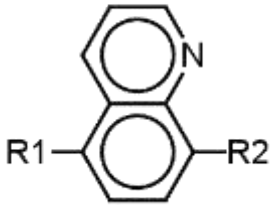	-14.41	-13.26
24	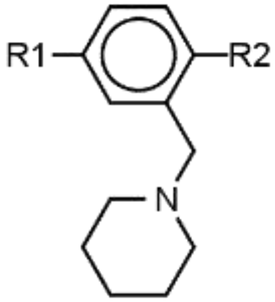	-12.14	-12.42
25	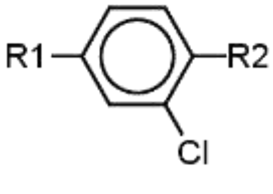	-15.00	-14.99

Table S2. Best docking scores of **7a** analogues for individual receptors. Compound numeration as in Table S1 (core 1).

Entry	3C6T	3DRP	4H4M	2YNG
1	-14.82	-15.31	-14.69	-14.93
2	-14.92	-15.48	-14.25	-15.22
3	-13.22	-15.53	-14.04	-15.65
4	-15.12	-15.48	-14.72	-15.26
5	-15.50	-15.42	-14.03	-14.21
6	-14.93	-14.77	-14.49	-15.12
7	-16.14	-15.80	-14.68	-16.17
8	-19.62	-14.90	-18.42	-15.33
9	-13.98	-15.50	-14.09	-14.65
10	-13.38	-15.78	-14.44	-15.34
11	-13.50	-12.96	-14.02	-14.24
12	-11.90	-12.80	-14.18	-14.66
13	-14.16	-14.68	-14.75	-14.84
14	-13.86	-15.34	-13.64	-15.03
15	-14.97	-15.37	-14.81	-15.24
16	-14.69	-13.70	-13.96	-14.66
17	-15.21	-15.27	-14.33	-15.30
18	-14.79	-14.69	-13.75	-14.88
19	-13.03	-13.56	-12.97	-13.44

20	-14.94	-15.13	-14.18	-14.43
21	-14.86	-15.74	-14.38	-15.45
22	-14.83	-14.85	-14.51	-15.19
23	-14.32	-14.40	-13.76	-15.16
24	-11.70	-11.93	-12.39	-12.56
25	-15.28	-15.39	-14.25	-15.09

Table S3. Best docking scores of **8a** analogues for individual receptors. Compound numeration as in Table S1 (core 2).

Entry	3C6T	3DRP	4H4M	2YNG
1	-14.93	-14.88	-14.58	-15.00
2	-14.86	-14.87	-14.47	-15.36
3	-13.23	-13.23	-14.95	-15.17
4	-15.39	-15.35	-14.86	-15.61
5	-15.16	-15.05	-13.46	-13.98
6	-15.09	-14.27	-14.27	-13.34
7	-16.64	-13.95	-14.09	-15.90
8	-18.40	-14.83	-18.09	-15.08
9	-14.93	-13.04	-15.34	-15.55
10	-15.16	-14.75	-13.84	-14.66
11	-13.50	-14.56	-14.21	-13.10
12	-12.72	-12.05	-14.11	-14.67
13	-15.59	-14.45	-14.73	-14.79
14	-14.73	-14.96	-14.73	-15.34
15	-14.77	-15.10	-14.50	-15.39
16	-13.66	-13.45	-13.41	-14.16
17	-15.45	-14.83	-13.57	-15.31
18	-15.11	-14.69	-14.60	-14.92
19	-13.28	-13.20	-13.58	-13.14

20	-15.45	-14.70	-14.30	-14.71
21	-15.37	-15.14	-15.09	-14.84
22	-15.53	-14.81	-12.98	-15.24
23	-15.30	-12.86	-14.11	-10.77
24	-12.81	-12.38	-13.44	-11.05
25	-15.20	-14.64	-14.98	-15.16

Table S4. Best docking scores for compounds **1-5, 7a-g** and **8a-f** for individual receptors. Compounds **1-5** are docked to receptors from their native PBD structures.

Compound	3C6T	3DRP	4H4M	2YNG
**1**	-	-	-16.21	-
**2**	-14.35*			
**3**	-14.83	-	-	-
**4**	-	-12.99	-	-
**5**	-	-	-	-14.97
**7a**	-14.82	-15.31	-14.69	-14.93
**7b**	-14.54	-14.86	-14.36	-14.48
**7c**	-14.86	-14.82	-14.74	-14.62
**7d**	-14.40	-14.66	-14.31	-14.20
**7e**	-19.62	-14.90	-18.42	-15.33
**7f**	-14.92	-15.48	-14.25	-15.22
**7g**	-15.12	-15.48	-14.72	-15.26
**8a**	-14.93	-14.88	-14.58	-15.00
**8b**	-14.69	-14.58	-14.69	-14.98
**8c**	-18.40	-14.83	-18.09	-15.08
**8d**	-14.86	-14.87	-14.47	-15.36
**8e**	-15.39	-15.35	-14.86	-15.61
**8f**	-19.52	-14.74	-16.91	-15.18

* Docked to 4NCG receptor

Table S5. Calculated octanol/water partition coefficients[a]

Compound	logD[b]
**7a**	1.57
**7b**	2.32
**7c**	1.57
**7d**	0.97
**7e**	3.05
**7f**	2.11
**7g**	2.11
**8a**	1.57
**8b**	1.57
**8c**	3.05
**8d**	2.11
**8e**	2.11
**8f**	3.05
NVP	2.49
RPV	5.47

[a] Computed using Marvin Sketch[5] with default weighted method

[b] pH = 7.4, NaCl concentration = 0.14 mmol/L

References

1. Tucker TJ, Sisko JT, Tynebor RM, Williams TM, Felock PJ, Flynn JA, Lai M, Liang Y, McGaughey G, Liu M, Miller M, Moyer G, Munshi V, Perlow-Poehnelt R, Prasad S, Reid JC, Sanchez R, Torrent M, Vacca JP, Wan B-L, Yan Y. Discovery of 3-{5-[(6-Amino-1H-pyrazolo[3,4-b]pyridine-3-yl)methoxy]-2-chlorophenoxy}-5-chlorobenzonitrile (MK-4965): A Potent, Orally Bioavailable HIV-1 Non-Nucleoside Reverse Transcriptase Inhibitor with Improved Potency against Key Mutant Viruses. J Med Chem 2008;51:6503–6511.
2. Tucker TJ, Saggar S, Sisko JT, Tynebor RM, Williams TM, Felock PJ, Flynn JA, Lai MT, Liang Y, McGaughey G, Liu M, Miller M, Moyer G, Munshi V, Perlow-Poehnelt R, Prasad S, Sanchez R, Torrent M, Vacca JP, Wan BL, Yan Y. The design and synthesis of diaryl ether second generation HIV-1 non-nucleoside reverse transcriptase inhibitors (NNRTIs) with enhanced potency versus key clinical mutations. Bioorg Med Chem Lett 2008;18:2959-2966.
3. Bollini M, Domaoal RA, Thakur VV, Gallardo-Macias R, Spasov KA, Anderson KS, Jorgensen WL. Computationally-Guided Optimization of a Docking Hit to Yield Catechol Diethers as Potent Anti-HIV Agents. J Med Chem 2011;54:8582−8591.
4. Saggar SA, Sisko JT, Tucker TJ, Tynebor RM, Su D-S, Anthony NJ. HIV Reverse Transcriptase Inhibitors. 2007; US patent 2007/0021442 A1.
5. Marvin 6.2.2, ChemAxon, Budapest, Hungary, 2014.
